# Supplementary material for: miR-10 and Its Negative Correlation with Serum IL-35 Concentration and Positive Correlation with STAT5a Expression in Patients with Rheumatoid Arthritis
Source: Int J Mol Sci. 2022 Jul 18;23(14):7925. doi: 10.3390/ijms23147925 (PMC9317037; doi:10.3390/ijms23147925)
Supplement: Supplementary file 1 [file ijms-23-07925-s001.zip › ijms-1768133-supplementary.pdf]

Supplementary materials

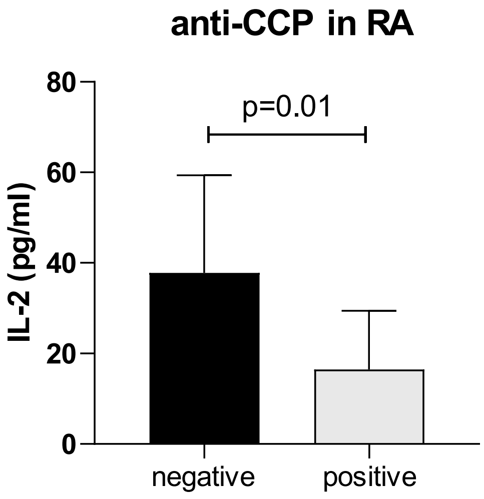

Figure S1. Serum IL-2 level in RA patients with positive and negative anti-CCP results.

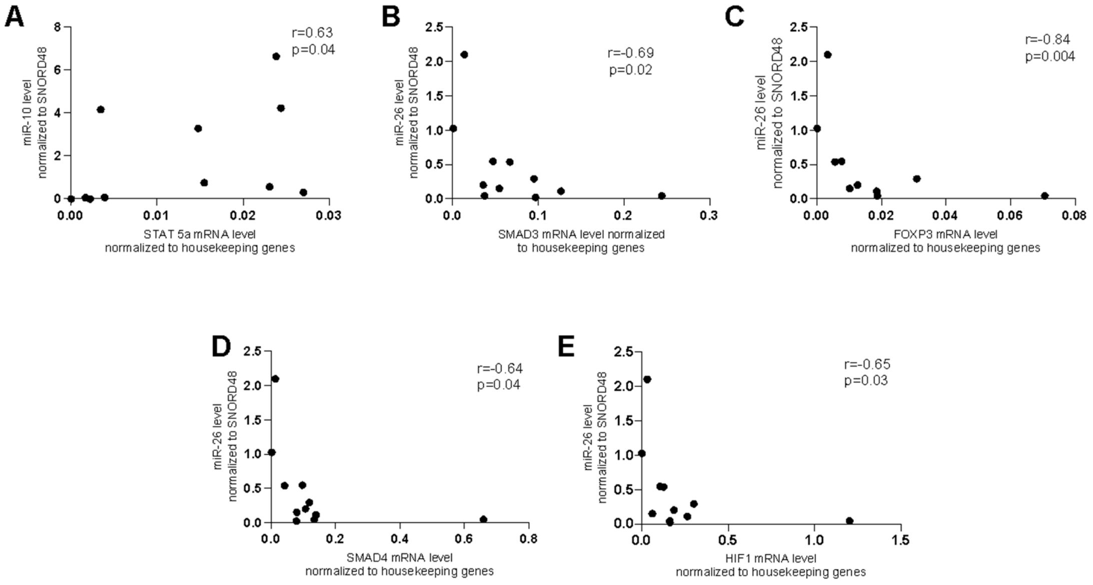

Figure S2. Correlation between miR-26 level in serum and analyzed expression of transcriptional factors in whole blood in RA patients.

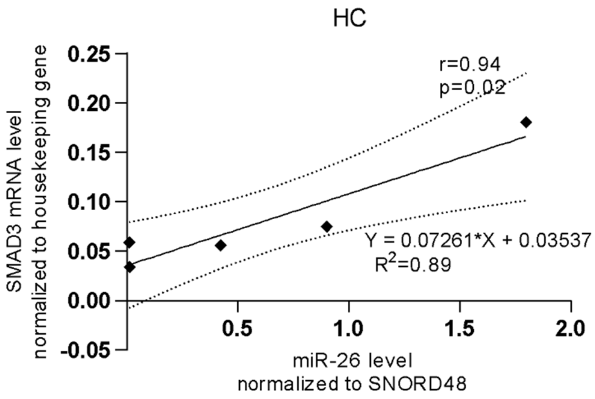

Figure S3. Correlation between miR-26 level in serum and *SMAD3* expression in whole blood in healthy controls (HC).

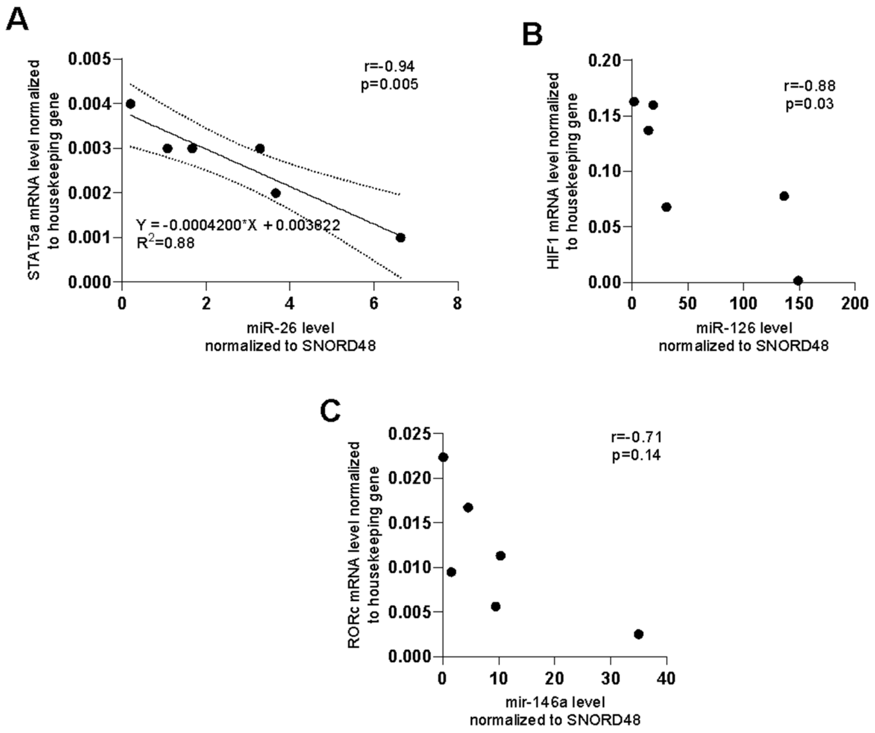

Figure S4. Pearson correlation between miR-26 level in serum and *STAT5a* expression in whole blood in OA patients (**A**). Spearman correlation between miR-126 in serum and *HIF1* mRNA level in whole blood (**B**) and miR-146a in serum and *RORc* mRNA level in whole blood (**C**) in OA patients.
